# The Interplay Between Dietary Choline and Cardiometabolic Disorders: A Review of Current Evidence

**DOI:** 10.1007/s13668-024-00521-3

**Published:** 2024-03-01

**Authors:** Natalia G. Vallianou, Dimitris Kounatidis, Sotiria Psallida, Fotis Panagopoulos, Theodora Stratigou, Eleni Geladari, Irene Karampela, Dimitrios Tsilingiris, Maria Dalamaga

**Affiliations:** 1 Department of Internal Medicine, Evangelismos General Hospital, 45-47 Ipsilantou str, Athens, Greece; 2 Department of Biological Chemistry, Medical School, National and Kapodistrian University of Athens, 75 Mikras Asias str, Athens, Greece; 3 Department of Internal Medicine, Hippokration General Hospital, 114 Vassilissis Sofias str, Athens, Greece; 4 Department of Microbiology, KAT General Hospital of Attica, 2 Nikis str, Athens, Greece; 5 Department of Endocrinology and Metabolism, Evangelismos General Hospital, 45-47 Ipsilantou str, Athens, Greece; 6 2nd Department of Critical Care, Medical School, University of Athens, Attikon General University Hospital, 1 Rimini str, Athens, Greece; 7 First Department of Internal Medicine, University Hospital of Alexandroupolis, Democritus University of Thrace, Dragana, 68100 Alexandroupoli, Greece

**Keywords:** Alzheimer’s disease, Cardiovascular disease, Cognition, Choline, Chronic kidney disease, Diet, Gut microbiota, Non-alcoholic fatty liver disease

## Abstract

**Purpose of Review:**

Choline is an essential nutrient for human health and cellular homeostasis as it is necessary for the synthesis of lipid cell membranes, lipoproteins, and the synthesis of the neurotransmitter acetylcholine. The aim of this review is to analyze the beneficial effects of choline and its significance in cellular metabolism and various inflammatory pathways, such as the inflammasome. We will discuss the significance of dietary choline in cardiometabolic disorders, such as non-alcoholic fatty liver disease (NAFLD), cardiovascular disease (CVD), and chronic kidney disease (CKD) as well as in cognitive function and associated neuropsychiatric disorders.

**Recent Findings:**

Choline deficiency has been related to the development of NAFLD and cognitive disability in the offspring as well as in adulthood. In sharp contrast, excess dietary intake of choline mediated via the increased production of trimethylamine by the gut microbiota and increased trimethylamine-N-oxide (TMAO) levels has been related to atherosclerosis in most studies. In this context, CVD and CKD through the accumulation of TMAO, p-Cresyl-sulfate (pCS), and indoxyl-sulfate (IS) in serum may be the result of the interplay between excess dietary choline, the increased production of TMAO by the gut microbiota, and the resulting activation of inflammatory responses and fibrosis.

**Summary:**

A balanced diet, with no excess nor any deficiency in dietary choline, is of outmost importance regarding the prevention of cardiometabolic disorders as well as cognitive function. Large-scale studies with the use of next-generation probiotics, especially *Akkermansia muciniphila* and *Faecalibacterium prausnitzii*, should further examine their therapeutic potential in this context.

## Introduction

Choline or 2-hydroxyethyl-trimethyl-ammonium salt is considered an essential nutrient, as only a small proportion can be synthetized by endogenous processes in the human body [1]. Due to the small amounts of de novo biosynthesis of choline, humans should receive choline from their diet. Eggs, red meat, milk, and cheese are the richest in choline dietary products. Choline is necessary for the function of various molecular pathways. In particular, it is prerequisite for the synthesis of the cells’ lipid membrane in the form of phosphatidylcholine and sphingomyelin. In addition, it is the precursor of the neurotransmitter acetylcholine, thus, being implicated in central nervous system (CNS) development and evolution throughout lifetime [1, 2]. Furthermore, choline is oxidized to betaine, which, in turn, contributes to the synthesis of S-adenosyl-methionine (SAM). SAM is the major methyl group donor in the human body. In this context, SAM plays a pivotal role in epigenetic DNA methylation and modulation [3, 4].

The Institute of Medicine suggested that choline is an essential nutrient in 1998, while in 2016, the European Food Safety Authority (EFSA) set dietary reference values for choline [5, 6]. According to the Institute of Medicine, daily adequate intakes (AIs) for adults are 550 mg for males and 425 mg for females, respectively [7]. Nevertheless, the EFSA in 2016 set the daily AIs to 400 mg for adults, 480 mg for pregnant women, and 520 mg for lactating women [6].

Notably, although there is no doubt that choline is an essential nutrient, there are many parameters, which induce modulations regarding its metabolism in the human body. The type and amount of dietary choline intake and genetic factors as well as the gut microbiota all constitute distinct features that interact with each other to further influence serum choline’s levels. In this review, we will focus on the aforementioned factors and we will discuss the significance of dietary choline in cardiometabolic disorders, such as non-alcoholic fatty liver disease (NAFLD), cardiovascular disease (CVD), and chronic kidney disease (CKD) as well as in cognitive function and associated disorders. In addition, we will elaborate upon the preventive and therapeutic potential of dietary changes and probiotics administration in terms of beneficial outcomes regarding human health.

## Choline: The Interplay Between Host Factors and Gut Microbiota

### Dietary Choline and Host Factors Affecting Its Metabolism

Dietary choline is classified in either water-soluble, such as free-choline, phosphocholine, and glycerophosphocholine, and lipid-soluble, such as phosphatidylcholine and sphingomyelin [1, 8]. The water-soluble form via portal circulation reaches the liver, whereas the lipid-soluble form by means of the lymphatic system participates into chylomicron formation [9]. Differences in dietary sources of choline play a crucial role in terms of water-soluble and lipid-soluble choline. A simple paradigm is the content of water-soluble and lipid-soluble choline in milk. Maternal milk has a different impact on choline absorption than the frequently consumed pasteurized milk [1, 9]. It is noteworthy that consumption of eggs, which are a well-known source of choline, may result in increases in serum cholesterol among individuals who have polymorphisms in the ATP-binding cassette (ABC) transporters (*ABCG*)* 5* and *ABCG8* genes. The above-mentioned polymorphisms account for the increased levels of cholesterol due to enhanced cholesterol absorption in the intestine [10•, 11]. Furthermore, it has been suggested that differences in age, sex, and sex hormones, such as estrogens, may influence the individuals’ needs for dietary choline. In particular, postmenopausal women need larger amounts of dietary choline in order to avoid organ dysfunction attributable to lower dietary choline intake. Postmenopausal women have lower serum estrogen levels, which, in turn, may affect the expression of *phosphatidylethanolamine-N-methyltransferase* (*PEMT*) gene [12, 13]. *PEMT* is the gene responsible for the breakdown of phosphatidylcholine into choline, leading to de novo choline production in humans [12, 13]. Fischer et al. have demonstrated that polymorphisms in the *PEMT* gene (*rs12325817*) are also implicated in the vulnerability of women regarding organ dysfunction due to lower serum choline levels [12].

Overall, apart from the water-soluble and lipid-soluble amount in dietary choline, other host factors, such as age, sex hormones, or gene polymorphisms, are involved in the metabolism of choline.

### Choline and the Gut Microbiota

The microbiota refers to the sum of bacteria, archaea, viruses, and fungi inhabiting the human body. It has been estimated that there are trillions of microorganisms, approximately 10^13^ to 10^14^ in the human body. As the vast majority of microbiota in humans resides in the gut, we often use the term “gut microbiota” instead [14, 15]. Under normal circumstances, there is homeostasis between the human gut microbiota and its host. However, under the influence of various factors, such as senescence, drugs, especially antibiotics, Western diet, or sedentary lifestyle, the phenomenon of “dysbiosis” occurs. Dysbiosis characterizes the imbalance between the gut microbiota and its host and has been suggested to be implicated in several diseases [14–18]. Metabolic disorders, such as obesity, hypertension, diabetes mellitus type 2, and NAFLD, together with other diseases, such as cancer, have been suggested to be also mediated by dysbiosis [14–18].

Regarding choline, it is widely known that it is converted to trimethylamine (TMA) by the gut microbiota. In turn, TMA is oxidized to trimethylamine-N-oxide (TMAO) via mono-oxygenases in the liver, which is then released into the systemic circulation [19–21]. Increased serum TMAO levels have been related to the development of atherosclerosis. In particular, elevated TMAO may promote the formation of foam cells from macrophages. Recently, it has been documented that increased TMAO is associated with the polarization of macrophages from the M2 phenotype, which exerts anti-inflammatory effects to the M1 phenotype, which exhibits pro-inflammatory properties. This phenotypic switch from M2 to M1 may be a plausible explanation for the atherogenic features of TMAO [22•, 23–25, 26•, 27]. Increased serum levels of TMAO may be attributed to an excess in dietary choline intake and/or abundant production of TMAO by the gut microbiota. Indeed, alterations in the composition and function of gut microbiota seem to play a pivotal role on the amount of gut-produced TMAO [28–30].

## The Role of Dietary Choline in Cardiometabolic Disorders

### Choline and NAFLD

NAFLD poses a global public health issue, as its prevalence has recently been estimated approximately 32% worldwide [31]. Among patients with NAFLD, non-alcoholic steatohepatitis (NASH) will develop in up to 25%. NASH has been suggested as the most common rising cause of hepatocellular carcinoma [32, 33]. NAFLD results from an imbalance between the de novo lipogenesis in the liver and the lipid hepatic excretion, leading to fat accumulation. It is associated with various degrees of liver fat deposition. It is a metabolic disorder that ranges from simple hepatic steatosis to the addition of inflammation (NASH), the development of cirrhosis, and even hepatocellular carcinoma [32, 33]. For the development of NAFLD, the most dominant hypothesis is the “multi-hit hypothesis.” This consists of the involvement of various genetic and epigenetic factors, apart from nutritional aspects, hormonal components, the implication of adipose tissue, and the modulation of the gut microbiota [21, 32, 33].

Choline-deficient diet and choline-methionine-deficient diet have long been used in animal models to provoke NAFLD [34]. Choline is a widely known methyl group donor in humans. As such, choline is necessary for the biosynthesis of phosphatidylcholine in the liver. In turn, phosphatidylcholine is a prerequisite for the synthesis of very low-density lipoproteins (VLDL). A choline-deficient diet leads to the decreased synthesis of phosphatidylcholine in the liver and, thus, to the aggregation of triglycerides in liver cells. Therefore, lipid accumulation in the liver occurs resulting gradually in the development of NAFLD [35, 36].

**Fig. 1:**
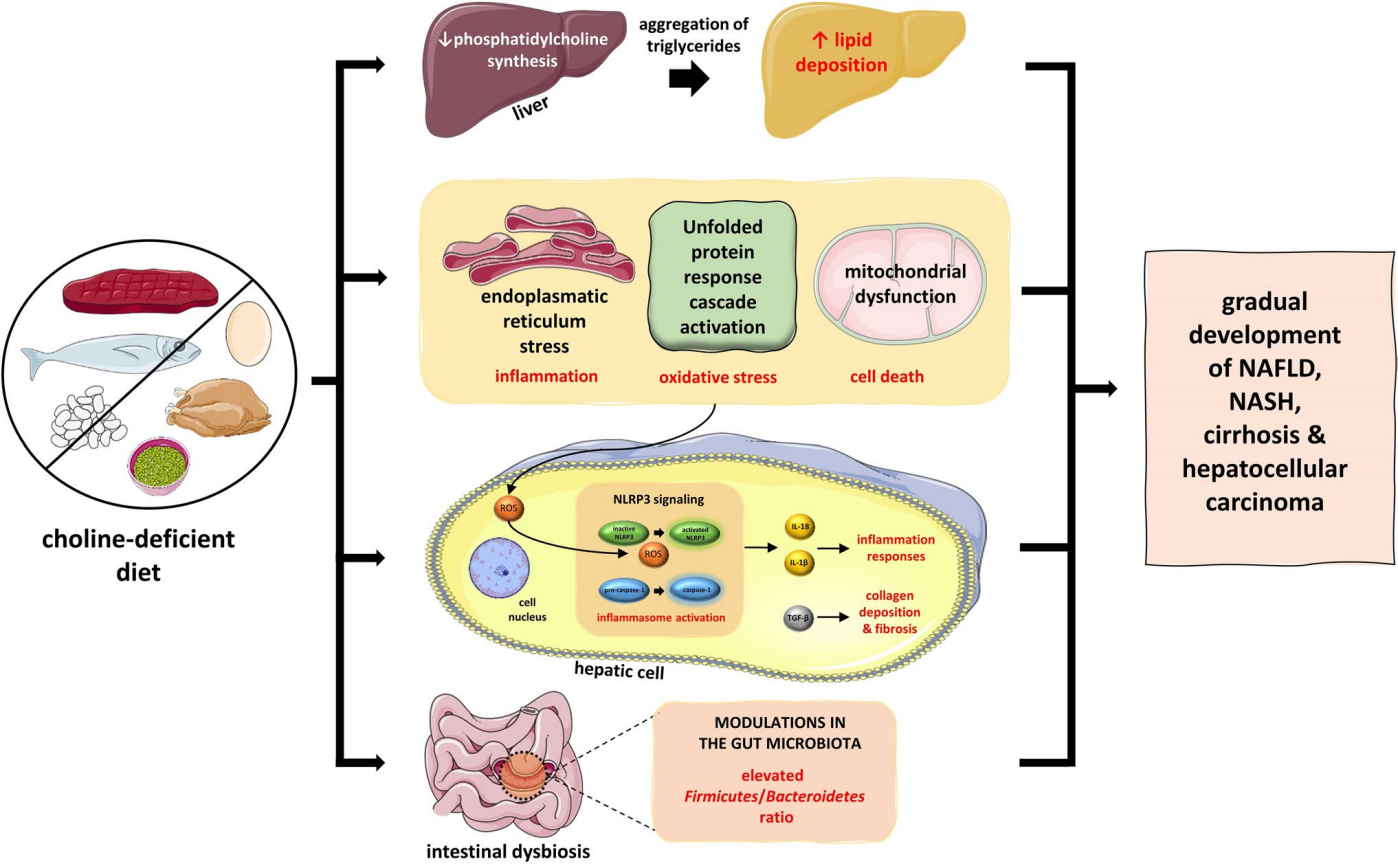
Dietary choline deficiency as a part of the “multi-hit hypothesis” in the development of NAFLD. Abbreviations: NAFLD, non-alcoholic fatty liver disease; NASH, non-alcoholic steatohepatitis; NLRP3, nucleotide-binding domain-like receptor protein 3; ROS, reactive oxygen species; TGF-β, transforming growth factor β. All free elements in the figure are originated from the free medical site http://smart.servier.com/ (accessed on January 1, 2024) by Servier licensed under a Creative Commons Attribution 3.0 Unported License

In addition, choline-deficient diet has been related to mitochondrial dysfunction due to endoplasmic reticulum (ER) stress [37, 38]. ER, among other functions, is responsible for protein maturation. Excess lipid deposition in the liver leading to hepatic steatosis is characterized by a subsequent enhancement in the needs for protein maturation by the ER. This increased burden cannot be satisfied, thus, accounting for unmet protein folding. The whole process results in ER stress, i.e., an abundance of unfolded proteins in the ER. Then, a cascade, which is widely known as unfolded protein response (UPR), is activated to maintain homeostasis. However, if the ER stress becomes chronic, this UPR cascade may lead to significant inflammation, increased oxidative stress, and even cell death [39, 40]. Therefore, while the aim of the UPR is to achieve cell homeostasis, chronic ER stress, such as in the case of NAFLD, may result in the activation of inflammatory responses [39, 40]. Inflammation is further augmented by the binding of reactive oxygen species (ROS) to the inflammasome (nucleotide-binding domain-like receptor protein 3) NLRP3. The inflammasome is a protein complex, which plays a crucial role in the pathogenesis of inflammation. More specifically, this binding of ROS to NLRP3 leads to the activation of the NLRP3. Activation of NLRP3 is achieved by a transformational change in the structure of NLRP3, which is attributed to the activation of caspase-1 from procaspase. In turn, the activated NLRP3 results in the release of various pro-inflammatory cytokines, especially interleukin (IL) 1β and IL-18 [41, 42]. This vicious cycle accounts for the development of inflammation seen in NASH, whereas the release of tumor growth factor β (TGF-β) is responsible for collagen deposition and the progression of NAFLD to cirrhosis [43, 44]. Figure 1 depicts the whole process, from chronic ER stress to the UPR, inflammation, and fibrosis in the context of NAFLD.

Apart from chronic ER stress and the UPR cascade, the activation of NLRP3, and the subsequent inflammatory responses, the gut microbiota is also deeply involved in the pathogenesis of NAFLD [45•]. In particular, using 16S rRNA sequencing techniques, it has been documented that there is an increase in *Bacteroidetes* and modulations in the abundance of *Firmicutes* among patients with NAFLD/NASH. In most of the studies regarding NAFLD/NASH, a decrease in the ratio of *Firmicutes/Bacteroidetes* (F/B) has been demonstrated [21]. Nevertheless, among patients with different stages of NAFLD/NASH, differences in the composition of the gut microbiota have been observed and the different stages of NAFLD may account for these reported discrepancies [21].

Overall, dietary choline deficiency by means of chronic ER stress, activation of the NLRP3, and modulations in the gut microbiota has been suggested to be part of the “multi-hit hypothesis” in the development of NAFLD. Even though NAFLD is a multifactorial process, it seems likely that a balanced diet, by avoiding deficiency or excess of choline intake, plays a key role in the prevention of NAFLD. Regarding therapeutic agents, it has been advocated that polyene phosphatidylcholine supplementation may offer a beneficial effect on NAFLD progression [46]. Notably, Zhang et al. have very recently reported significant improvement in the lipidomics and metabolomics in mice fed a choline-deficient diet, when treated with polyene phosphatidylcholine [47]. In particular, they documented changes in 14 lipids and 19 metabolites, in total [47]. Therefore, a balanced diet together with polyene phosphatidylcholine supplementation may represent novel and advantageous ways for the prevention and treatment of NAFLD.

### Choline and CVD

CVD, including heart disease, stroke, and peripheral artery disease, is the leading cause of death worldwide [48]. According to the CDC, 1 out of 5 deaths in the USA is attributed to CVD [49]. As already mentioned above, TMAO, a metabolite of choline derived in the gut, has been related to atherosclerosis and CVD risk [50–60]. TMA is produced directly from the gut microbiota and is further oxidized in the liver to TMAO. This oxidation is catalyzed by flavin mono-oxygenases, especially flavin mono-oxygenase 3 (FMO3).

#### Animal and Experimental Studies

In rodents, TMAO has been associated with an increased CVD risk. This association has been confirmed by fecal microbiota transplantation (FMT) from mice with CVD to previously health mice [61, 62]. The molecular mechanisms underlying the association between elevated serum TMAO levels and CVD remain largely unknown, so far. However, in 2021, Díez-Ricote et al. demonstrated a relationship between TMAO and specific microRNAs. miRNAs are small, non-coding RNA molecules, which are implicated in the regulation and expression of genes at the post-transcriptional level [63]. Astudillo and Mayrovitz have documented that TMAO levels increased the expression of miR-21-5p and miR-30c-5p. Through this molecular mechanism, TMAO is correlated to lipid metabolism, inflammation and, thus, to atherosclerosis [63]. They also identified that the aforementioned microRNAs modulated the expression of *Period Circadian Regulator 2* (*PER2*), their target gene [63]. Besides, more recently, in 2022, Díez-Ricote et al. have demonstrated another molecular pathway linking increased TMAO levels to atherosclerosis [64]. More specifically, they reported that TMAO may upregulate members of the miR-17/92 cluster, and this upregulation is associated with an enhancement in IL-12A. The release of IL-12A is strongly related to inflammation, while TMAO is also associated with increased levels of plasminogen activator inhibitor 1 (PAI-1) or SERPINE1, which in turn possess pro-thrombotic properties. Therefore, by documenting a relationship between TMAO, IL-12A, and PAI-1, Díez-Ricote et al. have managed to demonstrate another pathway of miR-17/92 cluster with inflammation and fibrin deposition in the atherogenic plaque [64].

#### Human Studies

Apart from animal models, there are many studies in humans as well. Díez-Ricote et al. have conducted a study among 20 male patients with metabolic syndrome who received lean donor FMT and found alterations in the composition of the gut microbiota, but not significant changes in TMAO levels [65]. On the contrary, Kanitsoraphan et al. have demonstrated an association of TMAO levels with CVD risk together with modifications in the gut microbiota [60]. In their meta-analysis, Smits et al. concluded that they did not find any significant relationship between dietary choline or betaine and incident CVD. However, they noted that further research should be performed regarding choline intake and CVD mortality [66]. In particular, in their meta-analysis, they found only two studies that met their inclusion criteria. One study by Meyer and Shea reported a positive association between dietary phosphatidylcholine intake and CVD mortality [67]. In sharp contrast, the second study by Zheng et al. did not confirm any association between dietary phosphatidylcholine intake and CVD mortality in a Japanese population [68]. However, Meyer and Shea concluded that the fact that only two studies were included in their meta-analysis and these studies had conflicting results is a severe limitation of their work. They pointed out the need for further large-scale studies in order to shed light upon the possible association between dietary choline intake and incident CVD or CVD mortality [69].

Although the results are inconclusive, most studies support the notion that TMAO levels are related to atherosclerosis and CVD. More specifically, TMAO levels may be associated with CVD regardless of other well-known CVD risk factors, such as serum low-density lipoprotein (LDL) cholesterol concentrations and chronic low-grade inflammation [70]. It is noteworthy that elevated serum TMAO levels may be predictive of increased CVD risk, even though other well-established CVD risk factors, such as lipid parameters, are within the normal range [71]. Moreover, TMAO levels are suggested to be related to the formation and progression of the atherosclerotic plaque [22•, 23–25, 26•, 27, 69, 70].

Nevertheless, very recently, Zhou et al. have enrolled 14,323 adults in the National Health and Nutrition Examination Survey (NHANES) between 2011 and 2016 and have found that a higher choline intake was related to a lower risk of CVD, especially stroke [71]. Although this is a rather unexpected finding, the authors conclude that further studies are needed to confirm or refute their findings [71].

Regarding nutritional aspects, a Western diet is rich in eggs and red meat, which are well-known sources of dietary choline. Thus, a Western diet is typically associated with increased TMAO production, whereas a healthier diet, such as the Mediterranean diet, has been inversely related to serum and urinary levels of TMAO [72]. Apart from the Mediterranean diet, probiotics, prebiotics, and their combination synbiotics might play a unique role in this context. The administration of probiotics, especially next-generation probiotics (NGP), such as *Akkermansia muciniphila*, has already been suggested to offer beneficial effects [73–75]. In particular, *Akkermansia muciniphila* has been documented to decrease the formation of abdominal aortic aneurysm, when administered in a mouse model. Furthermore, supplementation of rodents with *Akkermansia muciniphila* has resulted in an amelioration of the increased TMAO levels and blood pressure as well. It is noteworthy that Depommier et al. in their landmark study among humans have demonstrated that supplementation with this NGP has been a safe and well-tolerated method to improve parameters of the metabolic syndrome among overweight/obese patients [76].

Overall, although the debate regarding TMAO as a biomarker of incident CVD or CVD mortality is still on, there is no doubt that TMAO, as a product of dietary choline, will be a subject of ongoing research. Excess dietary choline intake, which may lead to increased serum TMAO levels through metabolism by the gut microbiota, should generally be avoided.

### Choline and CKD

CKD poses a serious public health problem affecting approximately 15% of the population in the USA, as has recently been estimated [77]. It is noteworthy that CVD accounts for the majority of mortality among patients with CKD [78]. TMAO is mainly excreted by the kidneys; therefore, in cases of CKD, serum TMAO levels are elevated. As already mentioned, increased TMAO levels have been associated with CVD. In particular, Go et al. have only recently documented a relationship between increased dietary choline intake and CKD-associated CVD [79]. Notably, they have demonstrated that excess choline intake by means of increased TMAO production results in decreased angiogenesis in the heart vessels. According to Xie et al., this reduction in the formation of new heart vessels is mediated by hypoxia-induced factor 1α (HIF-1α). More specifically, they documented that increased choline intake via a decrease in the release of HIF-1α may lead to ischemic cardiac disease in a mouse model of CKD-induced CVD [79]. In ischemic heart disease, angiogenesis plays a crucial role [80, 81]. The generation of new vessels in the heart through the release of compounds, such as HIF-1α, HIF-2α, vascular endothelial growth factor (VEGF), platelet-derived growth factor (PDGF), and angiopoieitin-2, is cardioprotective [82]. Therefore, this molecular mechanism of decreased production of HIF-1α as a result of excess choline intake may provide a plausible explanation for the CKD-induced CVD [79–82]. Very recently, Li et al. in their meta-analysis have documented a relationship between serum TMAO levels and CVD as well as all-cause mortality among patients with CKD [83].

**Fig. 2:**
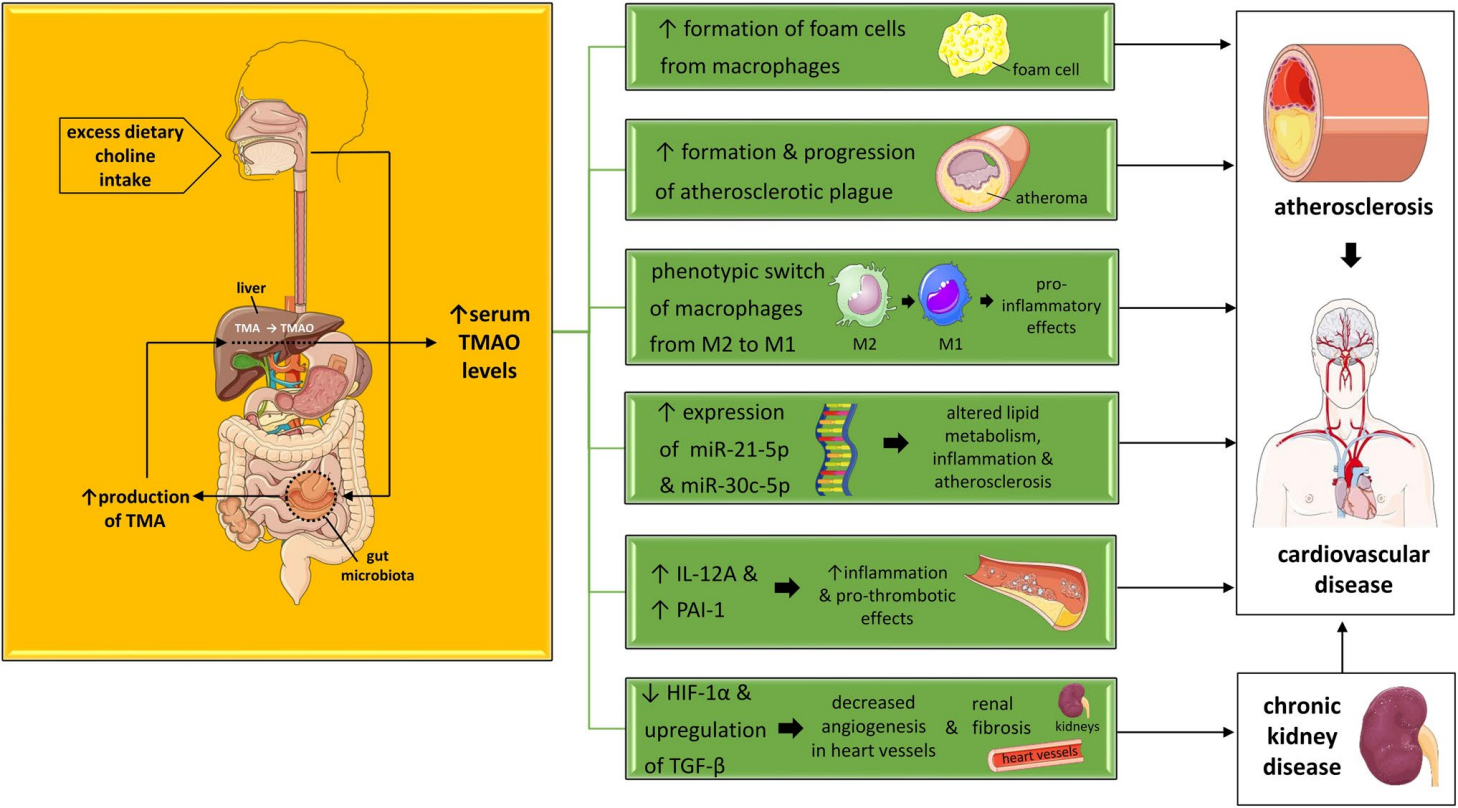
Main mechanisms explaining the association of excess choline intake with CVD and CKD. Abbreviations: HIF, hypoxia-induced factor; IL, interleukin; miRNAs, microRNAs; PAI-1, plasminogen activator inhibitor 1; TGF-β, transforming growth factor β; TMA, trimethylamine; TMAO, trimethylamine-N-oxide. All free elements in the figure are originated from the free medical site http://smart.servier.com/ (accessed on January 1, 2024) by Servier licensed under a Creative Commons Attribution 3.0 Unported License

Apart from CKD-induced CVD, choline has been suggested to be related to CKD per se. Serum TMAO levels are elevated among CKD patients due to the decreased renal clearance. In addition, gut microbiota alterations have been documented to occur in patients with CKD [18, 84]. The brain-gut-kidney axis supports the notion that changes in the composition of the gut microbiota are involved in the increased production and decreased renal elimination of uremic toxic metabolites, such as TMAO, p-Cresyl-sulfate (p-CS), and indoxyl-sulfate (IS) [18, 84]. Figure 2 describes the main mechanisms through which CVD and CKD are suggested to be associated with choline intake.

In animal models, rodents that had been fed with choline or TMAO were documented to develop renal fibrosis and renal impairment [85]. Besides, in another study, Qu et al. have fed mice with choline or TMAO and have reported an upregulation of the transforming growth factor β (TGF-β), which is a widely known factor contributing to fibrosis [86]. This upregulation of TGF-β in the kidneys has been attributed to an increase in the phosphorylation of SMAD3, which is involved in the SMAD3/TGF-β molecular pathway [86]. SMAD3 is a protein transducer of the activation of TGF-β, resulting in increases in the production of TGF-β and subsequently in renal fibrosis [86]. Xie et al. have also demonstrated that mice fed with choline displayed increased renal fibrosis, which was reduced with the use of antibiotics. Antibiotic usage resulted in alterations in the composition of the gut microbiota and, thus, in the reduction of the microbiota-derived TMAO levels [87]. Notably, Zhang et al. reported an improvement in renal fibrosis in mice with CKD, when administered an inhibitor of TMAO, iodomethylcholine [23].

Despite the fact that most studies on TMAO and CKD studies have been performed in animal models, there are a few related human studies as well. Tang et al. followed 521 patients with CKD for 5 years and found that increased TMAO levels were associated with a 2.8 times increased mortality rate [85]. Albeit the rarity of human studies, elevated serum TMAO levels have been associated with CKD [83, 88–92]. Interestingly, Mediterranean diet has not been documented to improve significantly CKD in one study. The authors concluded that the use of probiotics could be more beneficial in this context [93]. The role of NGPs, such as *Faecalibacterium prausnitzii*, is currently being further investigated [93]. Nevertheless, excess dietary choline in CKD patients should be avoided, as there seems to be a causative role of increased dietary choline intake and progression to CKD. Figure 2 shows the association between dietary choline intake and CKD and CVD.

## Dietary Choline, Cognitive Function, and Associated Disorders

Choline is a major element of the membrane component phosphatidylcholine and of the neurotransmitter acetylcholine [94]. Lately, there is a growing body of evidence advocating the maternal choline supplementation in order to improve cognitive ability in their offspring [95–98]. This advantageous supplementation has been suggested to act via an enhancement in the function of the hippocampus. Moreover, it has been documented to last throughout lifetime [95–98]. It is noteworthy that maternal choline supplementation is likely to protect from Alzheimer’s disease (AD) later in life as well [95–98]. It has been demonstrated that prenatal choline supplementation has been associated with increased levels of nerve growth factor (NGF), brain-derived neurotrophic factor (BDNF), VEGF, and insulin-like growth factor 2 (IGF2) in the hippocampus. All these factors seem to be implicated in the increased function of hippocampus and the amelioration in cognitive ability [96–99]. In addition, it has been shown that supplementation with choline during adulthood has been related to improved cognitive ability. Besides, choline supplementation has been associated with less white matter hyperintensity in magnetic resonance imaging, the latter being an imaging feature suggestive of AD [96]. Apart from the amelioration in cognitive ability, maternal choline intake has been associated with less neural tube defects, a better outcome in patients with Down and Rett syndromes, which are characterized by cognitive impairment and improved effects regarding autism and schizophrenia disorders [96, 97, 100]. Table 1 shows main studies in animal and humans that have been performed between 2013 and 2023 regarding dietary choline supplementation and neuropsychiatric disorders.

**Table 1:** List of main studies associating choline intake with neuropsychiatric disorders

**Research/year**	**Population, type of study**	**Main findings**	**Remarks**
***Animal studies***			
Ricceri et al., 2013 [101]	Adult MeCP2-308 mouse models of Rett syndrome	Post-natal choline supplementation improved neuromotor and behavioral indices in this MeCP2-308 mouse model	✓ Choline increased the level of neurotrophic factors in the hippocampus of these mice
Velazquez et al., 2013 [102]	Ts65Dn mouse model of Down syndrome	Early maternal choline intake improved the cognitive performance of the offspring	✓ Choline improved the neurogenesis of the hippocampus in the offspring
Kelley et al., 2014 [103]	Adult Ts65Dn mouse models of Down syndrome	Early maternal choline supplementation improved p75 neurotrophin receptor (p76NTR) and choline acetyltransferase	✓ Choline increased the choline acetyltransferase levels in the hippocampus
Ash et al., 2014 [104]	Ts65Dn mouse models of Down syndrome	Maternal choline supplementation increased basal forebrain neuron generation	✓ Maternal choline supplementation improved the size of the hippocampus in adult offspring
Borges et al., 2015 [105]	Mouse model of stroke	Following transient forebrain ischemia in mice, CA1 neurons of the hippocampus displayed prolonged survival	✓ Choline supplementation improved global ischemic brain area in mice
Langley et al., 2015 [106]	Mouse model of autism disorder, BTBR T + Itpr3tf/J (BTBR) mice	Maternal choline supplementation improved social skills in this model of autism spectrum disorder	✓ Maternal choline supplementation ameliorated behavior and decreased anxiety in this mouse model of autism disorder
Powers et al., 2017 [107]	Ts65Dn mouse models of Down syndrome	Maternal choline supplementation improved cognitive function in the offspring	✓ Choline improved attention and cognitive function by alterations in the nucleus basalis/substantia innominata of the offspring adults
Mellott et al., 2017 [99]	APPswe/PS1dE9 (APP.PS1) mouse model of Alzheimer’s disease	Maternal choline supplementation increased IGF-2, whereas a reduction in the number and the size of the amyloid plaques was reported	✓ Maternal choline supplementation increased the levels of choline acetyltransferase in the hippocampus of the mice
Jadavji et al., 2017 [108]	C57BI/6 wild-type mice, mouse model of stroke	After the mice suffered a stroke, the authors concluded that choline and B vitamin supplementation of mice resulted in decreases in the size of stroke	✓ Choline together with B vitamin supplementation reduced the area of stroke
Hurley et al., 2023 [109]	Mouse model of stroke, 10-month-old mice	Maternal dietary folic acid and choline deficiency were related to a bigger brain ischemic area	✓ Maternal choline and folic acid dietary deficiency were associated with larger brain volume lesions
Clementson et al., 2023 [110]	Mouse model of stroke, 3-month-old mice	Maternal dietary choline or folic acid deficiency was associated with decreased levels of betaine, HIF-1α, and caspase-3 in the offspring	✓ Maternal dietary choline deficiency was correlated to a worse motor outcome among offspring with stroke
Pull et al., 2023 [111]	Mouse model of stroke, 11.5-month-old mice	Maternal dietary choline or folic acid deficiency was associated with decreased brain blood flow in female offspring	✓ Maternal dietary choline or folic acid deficiency resulted in reductions in brain blood flow among female offspring with stroke. Early maternal supplementation may be beneficial regarding neurogenesis
***Human studies***			
Boeke et al., 2013 [112]	895 women and their offspring in the USA	With the use of the wide range assessment of memory and learning, the authors concluded that offspring visual memory at age 7 y.o. was improved with maternal’s choline supplementation	✓ Maternal choline intake was associated with a better memory of their offspring at 7 years of age
Lavery et al., 2014 [113]	409 Mexican–American women and their offspring	With the use of a FFQ, the authors concluded that there was a significant association between choline and betaine supplementation and lower risk for neural tube defects	✓ Choline and betaine supplementation prevented neural tube defects in their offspring

*FFQ* Food Frequency Questionnaire, *HIF-1α* hypoxia-induced factor 1 alpha, *IGF-2* insulin-like growth factor 2; *y.o.* years old

## Perspectives and Challenges

Choline metabolism in humans is a complex process. Apart from the amount of dietary choline intake, other parameters, such as its bioavailability, the gut microbiota composition, and genetic host factors, are involved. The gut microbiota depending on their microbial TMA lyases produce different amounts of TMA. TMA lyases are encoded by the *CutC/D* genes (chronic utilization cluster genes C and D), which are mainly responsible for TMA production [113]. Moreover, hormonal factors as well as host gene polymorphisms, such as *rs12325817* gene, are also implicated [12]. Therefore, a more personalized dietary approach depending on the individuals’ hormonal and health status, the knowledge of the gut microbiota composition and the genes implicated in choline metabolism, is necessary. To this end, multi-omics analysis may help identify important players and interactions. In particular, omics technologies comprise genomics (polymorphisms and other structural genetic variants), epigenomics (DNA methylation, histone modifications, microRNAs), metagenomics (gut microbiota composition), transcriptomics (RNA expression patterns), proteomics (protein profiles), and metabolomics (metabolite profiles), as well as the study of interactions with dietary factors. The field of microbiome research was revolutionized by using modern molecular methods and bioinformatics. The same holds true for lipidomics, i.e., the methodology of analyzing lipids, such as phosphatidylcholine and lysophosphatidylcholine. The combination of multiple omics represents an innovative holistic approach to provide a more integrated view of the molecular and physiological events underlying human disorders (including obesity, metabolic syndrome, NAFLD, CVD, and CKD) as well as for deciphering unique and specific metabolic phenotypes. Although more data are still necessary, it is expected that the incorporation of integrative omics may be useful not only for early diagnosis and risk prognosis but also for guiding tailored dietary treatments and prediction schemes [114–122]. As there are significant inter-individual variations, all the above-mentioned parameters should be considered to make the right decisions in an updated and personalized manner. However, there are many challenges that include the lack of robust and reproducible results due to methodological parameters, the elevated cost of omics methodologies, and the presence of high-dimensional data analyses and interpretation as well as ethical and regulatory issues.

## Conclusion

Choline is an essential nutrient for human health. Choline deficiency has been related to the development of NAFLD and cognitive disability in the offspring as well as in adulthood. In sharp contrast, excess dietary intake of choline mediated via the increased production of TMA by the gut microbiota and the subsequent increased levels of TMAO has been associated with atherosclerosis in most studies. In this context, CVD and CKD through the accumulation of TMAO, p-CS, and IS in serum may be the result of the interplay between excess dietary choline, the increased production of TMAO by the gut microbiota, and the resulting activation of inflammatory responses and fibrosis. Therefore, a balanced diet, with no excess nor any deficiency in dietary choline, is of outmost importance. Apart from a balanced diet, the potential benefit of NGPs, such as *Akkermansia muciniphila* and *Faecalibacterium prausnitzii*, should be further examined in large-scale randomized controlled studies.

## Data Availability

Not applicable.

## Abbreviations

ABC: ATP-binding cassette
AD: Alzheimer disease
BDNF: Brain-derived neurotrophic factor
CDC: Centers for Disease Control
CKD: Chronic kidney disease
CNS: Central nervous system
CVD: Cardiovascular disease
EFSA: European Food Safety Authority
ER: Endoplasmic stress
FMO3: Flavin mono-oxygenase 3
FMT: Fecal microbiota transplantation
HIF-1a: Hypoxia-induced factor 1a
IGF-2: Insulin-like growth factor 2
IL: Interleukin
IS: Indoxyl-sulfate
miRNAs: MicroRNAs
NAFLD: Non-alcoholic fatty liver disease
NASH: Non-alcoholic steatohepatitis
NGF: Nerve growth factor
NGP: Next-generation probiotic
NHANES: National Health And Nutrition Examination Survey
NLRP3: Nucleotide-binding domain-like receptor protein 3 inflammasome
PAI-1: Plasminogen activator inhibitor-1
pCS: P-Cresyl-sulfate
PDGF: Platelet-derived growth factor
PEMT: Phosphatidylethanolamine-N-methyltransferase
PER-2: Period Circadian Regulator 2
ROS: Reactive oxygen species
SAM: S-Adenosyl-methionine
TGF-β: Tumor growth factor β
TMA: Trimethylamine
TMAO: Trimethylamine-N-oxide
VEGF: Vascular endothelial growth factor
VLDL: Very low-density lipoproteins
UPR: Unfolded protein responses
